# Surgical techniques for degenerative cervical spine in Finland from 1999 to 2015

**DOI:** 10.1007/s00701-019-04026-9

**Published:** 2019-08-10

**Authors:** Anna Kotkansalo, Antti Malmivaara, Merja Korajoki, Katariina Korhonen, Ville Leinonen

**Affiliations:** 1 Division of Clinical Neurosciences, Department of Neurosurgery, Turku University Hospital, PB 52, 20521 Turku, Finland; 2 Faculty of Medicine, Department of Clinical Medicine, University of Turku, Turku, Finland; 3 Centre for Health and Social Economics, National Institute for Health and Welfare, Helsinki, Finland; 4 Orton Orthopaedic Hospital, Helsinki, Finland; 5 Welfare district of Forssa, Forssa, Finland; 6 Unit of Clinical Neuroscience, Neurosurgery, University of Oulu and Medical Research Center, Oulu University Hospital, Oulu, Finland; 7 Institute of Clinical Medicine – Neurosurgery, University of Eastern Finland, Kuopio, Finland; 8 Department of Neurosurgery, Kuopio University Hospital, Kuopio, Finland

**Keywords:** Incidence, Degenerative cervical spine, Operative techniques, Regional differences, Nationwide register study

## Abstract

**Purpose:**

The purpose of this study is to assess the trends and regional variations in the operative techniques used for degenerative or rheumatoid cervical spine disease in Finland between 1999 and 2015.

**Methods:**

The Finnish Hospital Discharge Register (FHDR) was searched for the data on all the primary operations for degenerative cervical spine disease (DCSD) or rheumatoid atlanto-axial subluxation (rAAS). Operative codes were used to identify the patients from the FHDR and combined with diagnosis codes to verify patient inclusion. The patients were classified into three groups: anterior cervical decompression and fusion (ACDF), posterior decompression and fusion (PDF) and decompression.

**Results:**

A total of 19,701 primary operations were included. The adjusted incidence of ACDF rose from 6.5 to 27.3 operations/100,000 adults. ACDF became the favoured technique in all the diagnostic groups except AAS, and by 2015, ACDF comprised 84.5% of the operations. The incidence of PDF for DCSD increased from 0.2 to 0.7/100,000 people. Solely decompressive operations declined from 13.7 to 4.0 operations/100,000 people. The regional differences in the incidence of operations were most marked in the incidence of ACDF, with overall incidences ranging from 11.2 to 37.0 operations/100,000. The distribution of the operative techniques used varied as well.

**Conclusions:**

Between 1999 and 2015, the operative techniques used for DCSD changed from prevalently decompressive to utilising ACDF in 68.8 to 91.0% of the operations, depending on the treating hospital. ACDF became the most commonly applied technique for all degenerative diagnoses except AAS.

**Electronic supplementary material:**

The online version of this article (10.1007/s00701-019-04026-9) contains supplementary material, which is available to authorized users.

## Introduction

The rate of surgery for degenerative cervical spine disease (DCSD) increased by 74% in Norway between 2008 and 2014 [15] and in the United States (US) by almost 150% between 1990 and 2013 [17, 24]. In the US, anterior cervical discectomy and fusion (ACDF) became the mainstay of treatment already in the late 1990s [24]. After 2011, the incidence of surgery for DCSD as well as the use of ACDF has declined slightly [17]. Recently, the incidence of posterior decompression and fusion (PDF) has increased steeply [17, 19, 23, 24]. The techniques applied vary between the regions in the US [3, 33] as well as internationally [8].

Our aim was to investigate the trends and the regional differences in the techniques utilised in the surgical treatment of the different degenerative or rheumatoid cervical spine conditions in Finland. The comprehensive administrative records enabled the reliable inclusion of every primary operation performed between 1999 and 2015.

## Materials and methods

### Study design and data sources

The PERFECT Cervical Spine database retrospectively combines data from the Finnish Hospital Discharge Register (FHDR), the Cause of Death Register and the registers of the Social Insurance Institute (SII) of Finland to include all the operations performed in Finland from 1999 to 2015 for degenerative or rheumatoid (RA) cervical spine disease. All of the administrative registries mentioned above utilise personal identity codes (PICs), which allow the data to be linked reliably on an individual level. The PICs also allow differentiation between the primary operations and the reoperations. The methods of the database construction and the data purification have previously been elucidated in detail [14]. The coverage and the accuracy of the data in the FHDR has previously been found to be good [29].

The Ethics Committee of the National Institute for Health and Welfare approved the creation of the PERFECT Spine database (THL 496/6.02.00/2011), and combining the data from the administrative registries was approved by the respective authorities. All the data in the PERFECT Spine database was acquired anonymized and the patients were not contacted. Therefore, informed consent was not required. The article was constructed in adherence with the STROBE guidelines.

### Study setting and patients

The patients were identified from the FHDR by using the Finnish version of the Nordic Medico-Statistical Committee Classification of Surgical Procedures (NOMESCO, http://urn.fi/URN:ISBN:978-952-245-858-2) operative codes as described previously [14]. The use of the operative code for anterior cervical decompression and fusion changed from ABC30 to NAG40/NAG41 during the years 2005 and 2006 in Helsinki University Hospital (personal communication), but in the other four university hospitals, the codes were used consistently over the period studied (personal communication). The primary and secondary operative codes were cross-linked with a diagnostic code from the 10th revision of the World Health Organization International Classification of Diseases (WHO ICD-10, the 2016 version) consistent with degenerative or rheumatoid cervical spine disease (http://urn.fi/URN:NBN:fi-fe201205085423) [14]. The analysis included every patient aged 18 years or older, residing in mainland Finland and with a WHO ICD-10 code consistent with degenerative or rheumatoid cervical spine disease. The patients with an ICD-10 code consistent with cancer, inflammatory spondylitis other than RA, other secondary spondylarthropaties, osteoporotic fracture, congenital spinal deformity, osteochondrodysplasia or trauma as an indication for surgery were excluded from the study, as well those patients with a previous cervical spine operation after 1986.

The patients were classified into five diagnostic and three procedure groups based on the diagnostic and the operative codes as described previously [14]. Only primary operations were included in the analysis. The comorbidity data was collected from the administrative registries mentioned above as well as the SII registers by using the special medication reimbursement codes and by the anatomical therapeutic chemical codes as reported previously [14].

### Statistical analyses

The incidence of surgery for each hospital was calculated based on the adult population (aged 18 years or older) of its referral area. The population characteristics were described with proportions, means and standard deviations. The measures of incidence were standardized for age and sex by the indirect method of standardization by comparing the ratio of operated patients to those expected using the mean of the entire adult population of mainland Finland between 1999 and 2015 as the reference. Statistical significance testing was not used as data is presented for the entire population rather than a sample of the population.

## Results

### Patients

There were 19,701 patients identified from the FHDR after the data purification who had undergone a primary cervical spine operation for degenerative or rheumatoid cervical spine disease. The mean age of the patients was 53.3 ± 11.4 years, and 44.4% of the patients were female. The patients operated by PDF were older and more frequently female in comparison with the other two technique groups. The patient demographics are detailed in Table 1.

**Table 1:** The description of the patients operated for degenerative or rheumatoid cervical spine disease in Finland between 1999 and 2015

	Decompression	ACDF^a^	PDF^b^	All
Patients (N)	5990	13,101	601	19,701
Female (%)	40.6	45.1	67.2	44.4
Age, mean ± SD	57.1 ± 12.7	51.1 ± 9.9	62.8 ± 11.5	53.3 ± 11.4
Age group (%)				
18–44	16.0	25.4	6.3	22.0
45–60	46.9	59.3	29.3	54.6
61–75	27.5	14.0	51.1	19.3
Over 75	9.6	1.3	13.3	44.2
Comorbidities (%)				
Rheumatoid arthritis	4.5	3.6	58.6	5.5
Hypertension	39.1	32.7	51.1	35.2
Atrial fibrillation	5.5	3.2	10.5	4.1
Cardiac insufficiency	2.3	0.6	4.8	1.3
Coronary artery disease	10.2	5.9	12.6	7.4
Peripheral artery disease	2.8	0.8	3.8	1.5
Hypercholesterolemia	11.4	12.5	12.3	12.1
Diabetes	9.5	7.6	9.0	8.2
Uremia	0.4	0.1	1.3	0.2
Cancer	6.7	4.0	10.6	5.1
COPD^c^ or asthma	15.2	16.8	13.6	16.2
Dementia	1.3	0.2	1.3	0.6
Demyelinating or neurodegenerative disease	1.7	1.0	0.8	1.2
Parkinson’s disease	1.6	1.1	2.8	1.3
Epilepsy	2.0	1.6	2.3	1.8
Cerebrovascular disease	6.3	4.1	5.8	4.8
Depression	17.5	21.9	16.3	20.4
Other mental disorder	3.7	3.9	3.3	3.8
Alcohol/drug addiction	4.0	4.3	2.5	4.1
Arthrosis of the hip or knee	4.8	2.1	7.7	3.1
Arthrosis of the shoulder	0.1	0.4	0	0.3
Rotator cuff syndrome	5.3	8.7	5.3	7.6
Fibromyalgia	0.1	0.1	0	0.1
Hospital status (%)				
Public	92.3	96.3	91.5	94.9
Private	7.7	3.7	8.5	5.1

^a^*ACDF* anterior cervical decompression and fusion, ^b^*PDF* posterior decompression and fusion, ^c^*COPD* chronic obstructive pulmonary disease

### Operation techniques

ACDF, typically with a standalone cage, comprised 66.5% of all the operations, while posterior fusions were rare, only 3.1% of the operations. Of the posterior fusions, 48.4% were performed for rheumatoid atlanto-axial subluxation (rAAS). In 30.4% of the operations, only decompression was performed. Only 57 total disc replacement (TDR) operations were performed during the study period according to the operative codes used (0.4% of all the ACDF/TDR operations, 0.8% of the operations for disc protrusion). Corpectomy was only recorded as a procedure code for degenerative cervical spine four times during the entire study period; all the cases were for spinal canal stenosis (SCS) or myelopathy. Only two patients underwent a 360° fusion during the same hospitalization period.

A more detailed analysis of the different techniques is less reliable due to coding irregularities, as evidenced by the Supplementary Table S2. In three of the five hospitals (Kuopio, Oulu and Tampere) with more reliable data, anterior decompression without fusion was utilised in 1.0–7.3% of the operations, foraminotomy in 3.2–17.4% of the operations and laminectomy/laminoplasty in 6.2–17.4% of the operations, depending on the hospital. Anterior plating was included in 4.3–17.5% of the ACDF operations in these three hospitals. The data is provided in Supplementary Table S2.

The distribution of the operative techniques utilised in each diagnostic group is given in Table 2. Data on the specific operation techniques within the diagnostic groups is provided in Supplementary Table S3.

**Table 2:** The distribution of the patients into the procedure and the diagnostic groups, *N* (%)

Diagnosis group		Procedure group		
		Decompression only	Anterior decompression and fusion/total disc replacement	Posterior decompression and fusion
Intervertebral disc protrusion		1593 (8.1)	5333 (27.1)	0
Foraminal stenosis		1402 (7.1)	5384 (27.3)	88 (0.4)
Spinal canal stenosis		3004 (15.2)	2384 (12.1)	192 (1.0)
AAS^a^	Degenerative	0	0	30 (0.2)
	Rheumatoid	0	0	291 (1.5)

^a^*AAS* atlanto-axial subluxation

The overall incidence of ACDF was 18.3/100,000 people aged 18 years or older, decompression 8.6 and PDF 0.8/100,000 people. For degenerative cervical spine disease (DCSD) only (excluding rAAS), the overall incidence of PDF was 0.4/100,000 people.

### Trends over time

**Fig. 1:**
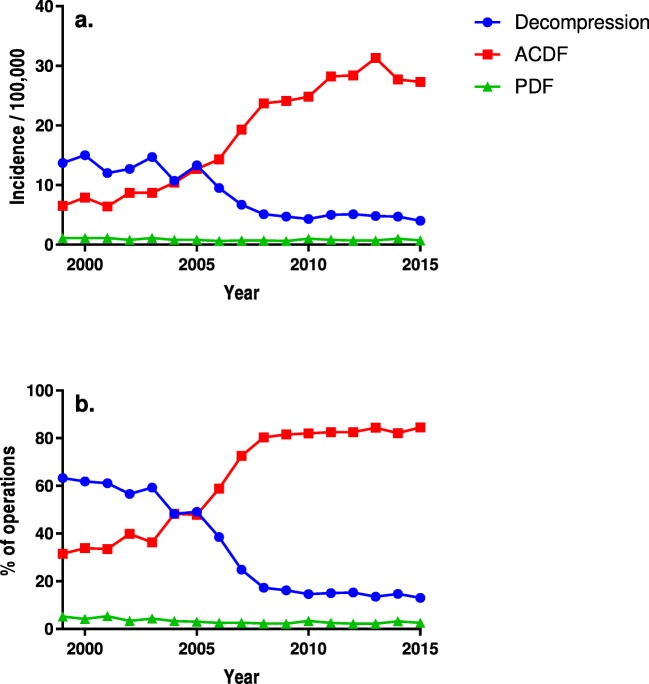
The annual age- and sex-adjusted incidences of operations (operations/100,000 people aged 18 years or older) in each technique group (**a**). The proportional use of each technique annually (**b**). *ACDF* anterior cervical decompression and fusion, *PDF* posterior decompression and fusion

The overall adjusted operation incidence rose from 20.7 to 31.7/100,000 people aged 18 years or older between 1999 and 2015 (the range in the annual incidences was 19.0–36.5/100,000 people). According to the operative codes, the incidence of ACDF rose by 320% (from 6.5 to 27.3 operations/100,000 people) over the 17-year period, while the incidence of decompressions diminished by 71% (from 13.7 to 4.0 operations/100,000 people) (Table 3). The overall incidence of PDF declined slightly, while the incidence of PDF for DCSD increased from 0.2 to 0.7/100,000 adults (Supplementary Table S1). The operation incidences and proportions are given in Table 3 and illustrated in Fig. 1 a and b.

**Table 3:** The adjusted incidences of the operations (operations/100,000 people aged 18 years or older) and the distribution of the operations between the technique groups

Year	Operations (*N*)	Adjusted operation incidences				Distribution of operations (%)		
		Decompression	ACDF^a^/TDR^b^	PDF^c^	Overall	Decompression	ACDF/TDR	PDF
1999	832	13.7	6.5	1.1	21.0	63.3	31.5	5.2
2000	945	15.0	7.9	1.1	23.6	61.9	33.9	4.2
2001	779	12.0	6.4	1.1	19.2	61.1	33.5	5.4
2002	899	12.7	8.7	0.8	22.0	56.6	39.9	3.4
2003	1002	14.7	8.7	1.1	24.3	59.3	36.3	4.4
2004	900	10.7	10.4	0.8	21.7	48.3	48.3	3.3
2005	1117	13.3	12.7	0.8	26.7	49.1	47.9	3.0
2006	1028	9.5	14.3	0.6	24.5	38.5	58.9	2.5
2007	1124	6.7	19.3	0.7	26.6	24.9	72.5	2.6
2008	1245	5.1	23.7	0.7	29.4	17.3	80.4	2.3
2009	1251	4.7	24.1	0.6	29.4	16.2	81.5	2.2
2010	1281	4.3	24.8	1.0	30.0	14.6	82.0	3.4
2011	1453	5.0	28.2	0.8	33.9	15.0	82.5	2.5
2012	1466	5.1	28.4	0.7	34.1	15.3	82.5	2.2
2013	1576	4.8	31.3	0.7	36.5	13.5	84.4	2.2
2014	1433	4.7	27.7	1.0	33.1	14.7	82.1	3.2
2015	1370	4.0	27.3	0.7	31.7	13.0	84.5	2.5
Overall	19,701	8.6	18.3	0.8	27.6	34.3	62.5	3.2

^a^*ACDF* anterior cervical decompression and fusion, ^b^*TDR* total disc replacement, ^c^*PDF* posterior decompression and fusion

**Fig. 2:**
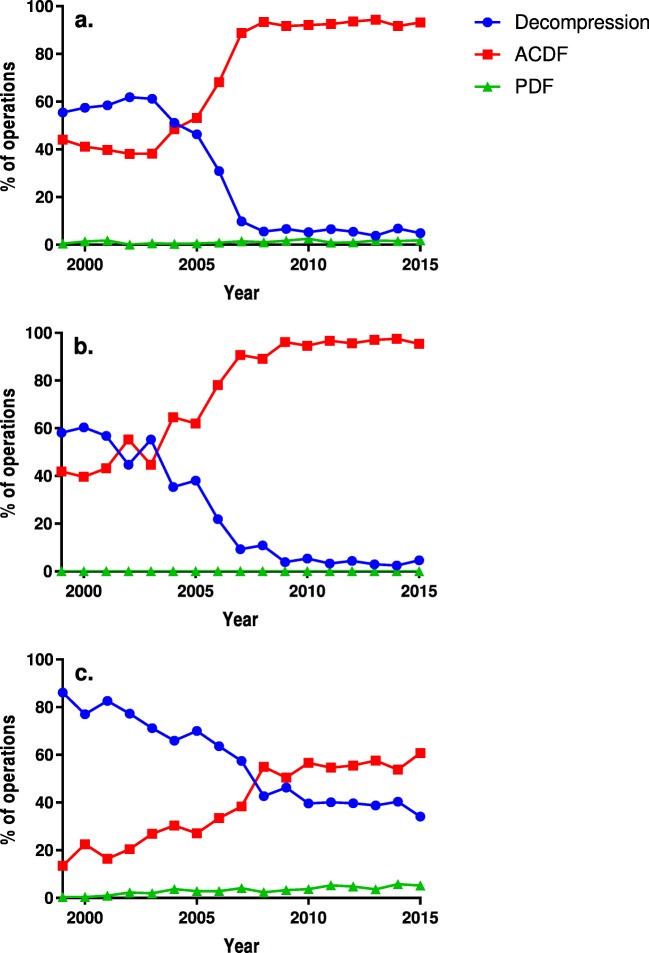
The proportional use of each technique annually for foraminal stenosis (**a**), disc protrusion (**b**) and spinal canal stenosis (**c**). *ACDF* anterior cervical decompression and fusion, *PDF* posterior decompression and fusion

ACDF became the most commonly applied technique in all the diagnosis groups except AAS (Fig. 2). Fusions for SCS increased from 13.8 to 65.9% of the operations; 92.5% of the fusions were ACDF. PDF increased in both the foraminal stenosis (FS) and the SCS groups, while foraminotomies decreased from 52.9 to 3.3% of the operations for FS. Unsurprisingly, only posterior fusion was used for AAS; a secondary decompression code was included in only 16 operations. The change in the operative techniques in each diagnostic group is depicted in Fig. 2. Especially the use of ACDF without anterior plating increased and the use of foraminotomy decreased, while the use of laminectomy remained fairly constant. Anterior decompressions where infrequent and declined rapidly with the rise of ACDF. The changes in the specific operative techniques over time are illustrated in Supplementary Fig. S4, as the use of specific operative codes has varied over time and between the hospitals, which influences the validity of the findings.

**Fig. 3:**
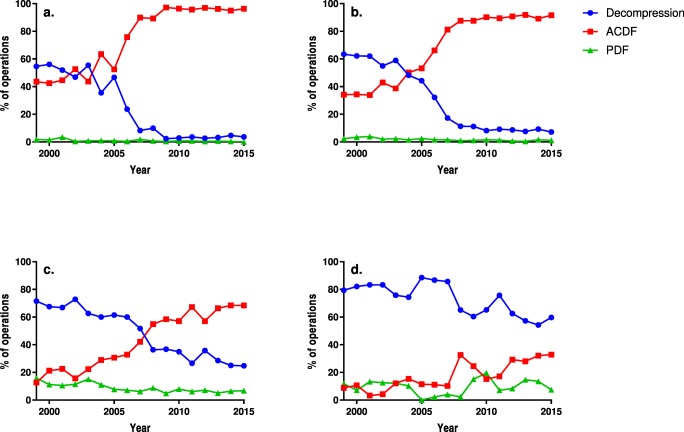
The proportional use of each technique annually in the 18–44- (**a**), 45–60- (**b**), 61–75-year-old age group (**c**) and in the over 75-year-old age group (**d**). *ACDF* anterior cervical decompression and fusion, *PDF* posterior decompression and fusion

The use of ACDF increased in every age group and became the most commonly applied technique in all but the oldest age group of patients: those over the age of 75 years. The proportional use of PDF decreased in all but the oldest age group. The changes in the operative techniques in each age group are depicted in Fig. 3.

### Regional differences

**Fig. 4:**
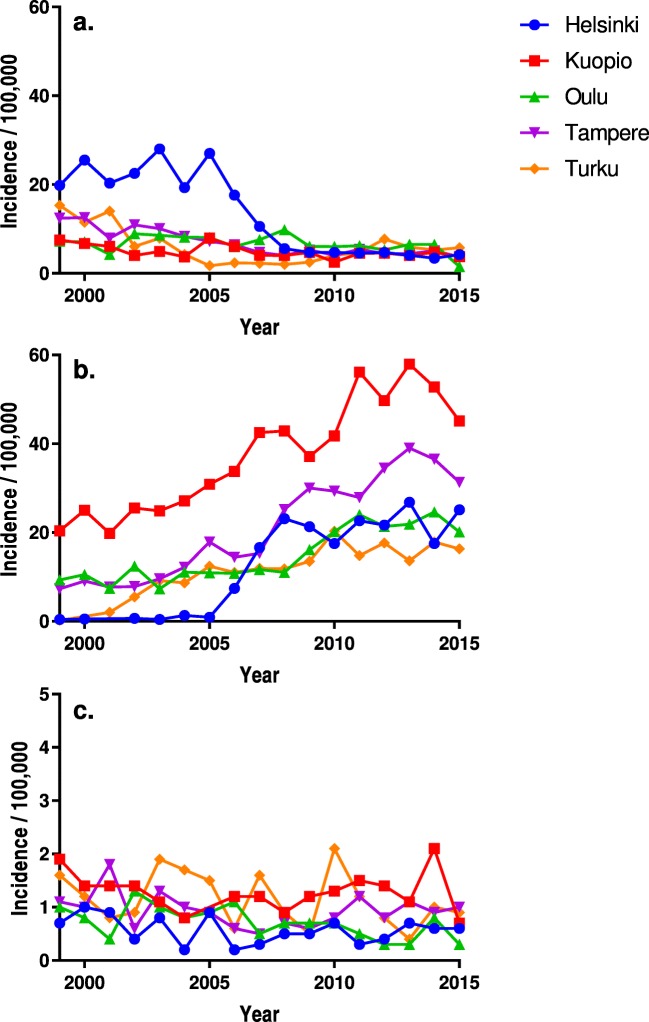
The adjusted annual incidences of the operations (operations/100,000 people aged 18 years or older) in different hospitals for decompression procedures (**a**), anterior cervical discectomy and fusion (**b**) and posterior decompression and fusion (**c**). Observe the difference in scaling between Figs. (**a**, **b**) and (**c**)

**Fig. 5:**
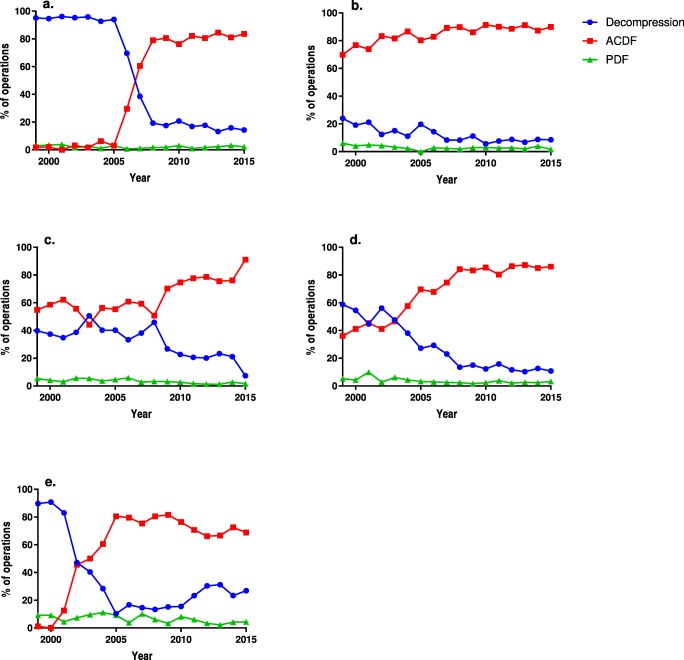
The proportional use of each technique annually in Helsinki (**a**), Kuopio (**b**), Oulu (**c**), Tampere (**d**) and Turku (**e**) university hospitals. *ACDF* anterior cervical decompression and fusion, *PDF* posterior decompression and fusion

The incidence and the proportional use of ACDF increased and decompression decreased in all the university hospitals at slightly differing time points (Figs. 4 and 5). The overall incidences of PDF varied from 0.6/100,000 at the lowest to 1.2/100,000 at the highest between the university hospitals, but PDF was infrequent overall and the differences in the incidences seem to have remained fairly stable (Table 4). Fusion was included in 73.1 to 92.6% of the operations in 2015, depending on the hospital.

**Table 4:** The adjusted annual operation incidences (operations/100,000 people aged 18 years or older) in each university hospital and technique group

	Decompression (anterior or posterior)					Anterior decompression and fusion/total disc replacement					Posterior decompression and fusion (PDF)					All the operations				
Year	Helsinki	Kuopio	Oulu	Tampere	Turku	Helsinki	Kuopio	Oulu	Tampere	Turku	Helsinki	Kuopio	Oulu	Tampere	Turku	Helsinki	Kuopio	Oulu	Tampere	Turku
1999	19.8	7.5	7.2	12.4	15.3	0.4	20.4	9.3	7.3	0.2	0.7	1.9	1.0	1.1	1.6	20.5	29.4	17.4	20.5	16.3
2000	25.5	6.7	7.1	12.6	11.5	0.5	25.0	10.5	9.1	0	1.0	1.4	0.8	1.0	1.2	26.7	32.8	18.3	22.5	12.1
2001	20.3	6.1	4.3	7.9	14.0	0	19.8	7.4	7.7	2.0	0.9	1.4	0.4	1.8	0.8	20.9	27.1	12.0	17.2	16.2
2002	22.5	4.0	8.9	10.9	6.1	0.7	25.5	12.4	7.8	5.5	0.4	1.4	1.3	0.6	0.9	23.5	30.8	22.5	19.1	12.4
2003	28.0	4.9	8.6	10.1	7.9	0.4	24.9	7.3	9.6	9.2	0.8	1.1	1.0	1.3	1.9	28.9	30.8	16.7	20.8	18.9
2004	19.3	3.7	8.2	8.3	4.3	1.3	27.1	11.1	12.2	8.7	0.2	0.8	0.8	1.0	1.7	20.7	31.6	19.9	21.4	14.6
2005	27.0	7.9	8.1	7.2	1.7	0.9	30.9	10.9	17.9	12.4	0.9	0	0.9	0.9	1.5	38.6	38.7	19.8	25.9	15.6
2006	17.6	6.1	6.0	6.4	2.4	7.4	33.8	10.8	14.4	11.0	0.2	1.2	1.1	0.6§	0.6	25.2	41.0	17.9	21.4	14.0
2007	10.6	4.1	7.6	4.7	2.3	16.6	42.5	11.7	15.3	11.9	0.3	1.2	0.5	0§.5	1.6	27.5	47.8	19.8	20.5	15.9
2008	5.6	4.0	9.8	4.0	2.0	23.1	42.9	11.0	25.2	11.8	0.5	0.9	0.7	0.7	0.9	29.2	47.8	21.5	29.9	14.6
2009	4.7	4.7	6.1	5.3	2.5	21.3	37.1	16.1	30.0	13.5	0.5	1.2	0.7	0.6	0.5	26.3	43.0	22.9	35.9	16.4
2010	4.7	2.5	6.0	4.1	4.0	17.5	41.8	20.2	29.3	20.3	0.7	1.3	0.7	0.8	2.1	22.8	45.5	26.9	34.1	26.3
2011	4.6	4.5	6.2	5.4	4.7	22.7	56.1	24.0	27.9	14.8	0.3	1.5	0.5	1.2	1.2	27.4	61.9	30.6	34.4	20.5
2012	4.7	4.6	5.3	4.4	7.7	21.7	49.7	21.4	34.5	17.7	0.4	1.4	0.3	0.8	0.8	26.6	55.6	26.9	39.4	26.1
2013	4.1	4.0	6.5	4.4	5.9	26.8	57.9	21.9	39.0	13.6	0.7	1.1	0.3	1.1	0.4	31.3	62.7	28.5	44.2	19.8
2014	3.4	4.8	6.5	5.1	5.3	17.5	52.8	24.6	36.5	17.9	0.6	2.1	0.8	0.9	1.0	21.3	59.6	31.7	42.3	23.9
2015	4.2	3.8	1.5	3.7	5.8	25.1	45.1	20.1	31.3	16.3	0.6	0.7	0.3	1.0	0.9	29.5	49.3	21.6	35.8	22.9
Mean	12.8	4.9	6.7	6.8	6.0	12.4	37.0	14.8	20.8	11.2	0.6	1.2	0.7	0.9	1.1	25.8	43.1	22.1	28.5	18.3

## Discussion

### Key results

According to the operative codes, the age- and sex-adjusted incidence of ACDF rose from 6.5 to 27.3 operations/100,000 adults (a 320% increase) between 1997 and 2015, and by 2008, ACDF was used in over 80% of the operations for DCSD. The incidence of solely decompressive procedures decreased by 71%, from 13.7 to 4.0 operations/100,000 people. PDF was applied in only 3.1% of the operations, and the overall incidence of PDF decreased from 5.2 to 2.5 operations/100,000 people; however, the use of PDF in treating DCDS increased from 0.2 to 0.7/100,000 people. The inclusion of fusion and the incidence of ACDF rose in all the age groups and all the diagnosis groups excluding AAS. ACDF became the most commonly applied technique in all the diagnosis groups except AAS and in all the age groups except the over 75-year-olds. The techniques used varied between the university hospitals, but a similar change from posterior decompressions to anterior decompression and fusion at slightly differing time points was evident in every hospital.

### Operative techniques

A shift in the operative techniques from solely decompressive procedures to anterior decompression and fusion was evident. According to the operative codes used, the incidence of ACDF rose from 6.5 to 31.3/100,000 people between 1999 and 2013 and decreased slightly after that. This is partly explainable by a 134% increase in the operations for FS [14], but ACDF also became the favoured technique in all the diagnosis groups except AAS. By 2015, ACDF accounted for 84.5% of the operations for DCSD. The use of ACDF increased in all the age groups. The change in the coding practices in Helsinki University Hospital has likely caused the rate of ACDF found, to be lower and the rate of decompression to be higher than it actually was during 1999–2006. Between 1999 and 2006, 36.3% (2006) to 82.7% (2003) (mean 68.6%) of the operations in Helsinki were coded ABC30, which equates to 12.5% (2006) to 32.6% (2003) (mean 24.3%) of all the operations during those years. The observed change in the operative techniques seems to have occurred in all the other hospitals as well but at slightly differing time points, and this likely reflects an actual change in practices despite the coding irregularities. The decline in decompressive operations may partly be explained by a decrease in foraminotomies, as the use of laminectomy appears to have remained consistent at around 10% of the operations (Supplementary Fig. S4). A similar shift from decompressions to ACDF occurred in the US already in the late 1990s [24]. After increasing for over three decades [19, 23, 24], the incidence of ACDF in the US has now decreased to 56.2/100,000 adults [17]; nevertheless, this is still almost double the recent incidence in Finland. Between 2001 and 2013, 80.6% of the operations for DCSD in the US were ACDF [17], while in Finland only 62.5% of the operations were ACDF between 1999 and 2015. However, in the last 5 years, ACDF accounted for 83.2% of the operations in Finland while PDF was used more infrequently than in the US (2.5% in Finland between 2011 and 2015 versus 7.5% PDF in the US between 2001 and 2013) [17].

While the incidence of solely decompressive procedures declined by 71% (from 13.7 to 4.0 operations/100,000 people), a 36% decrease (from 1.1 to 0.7 operations/100,000 people) in the incidence of posterior fusions was also observed in Finland. This can be explained by a decrease in surgery for rheumatoid AAS [14]; for DCSD, the incidence of PDF rose from 0.2 to 0.7 (Supplementary Table S1). This is in contrast to the findings from the US, where the rate of PDF in treating DCSD continues to increase [17, 19, 23, 24, 26]. In the US, the recent reported incidence of PDF, 7.81/100,000 adults [17], is six times higher than the incidence in Finland. In an analysis of all cervical fusion surgery in the state of New York, the increase in PDF surgery was the greatest for spondylosis, but the use of PDF or even circumferential fusion increased in all the degenerative diagnoses including disc disease [26]. There were no PDF surgeries performed for disc protrusion in Finland. The incidence of 360° fusion has risen by over 330% between 2001 and 2013 in the entire US [17]. In 2013, 91.3% of the operations in the US included fusion [17], while in Finland in 2015, 87.0% of the operations included fusion; however, 97.1% of the fusions were ACDF. Only two patients with 360° fusions were identified and were included in the PDF group.

TDR was rarely used, most likely because of the higher cost, more demanding surgical technique and the lack of unequivocal evidence on the long-term effectiveness of TDR in preventing adjacent segment disease [5, 7, 18, 22, 31]. Corpectomy was exceedingly rare; posterior decompressions were probably performed for SCS extending beyond the disc level or for multi-level SCS. Corpectomies may also have been coded inaccurately, prohibiting their identification.

The overall incidences of ACDF varied between the university hospitals by over 3-fold, from 11.2 to 37.0 operations/100,000 people. The variation in the incidences is mostly explained by the differences in the incidence of operations for DCSD overall and especially the operations for FS; the incidence of operations for FS varied from 1.6/100,000 to 21.1/100,000 between the hospitals [14]. For decompression and PDF, the incidences were also more than double in the highest incidence hospital compared with the lowest incidence hospital. Similar regional differences in the operative incidences and the techniques used have earlier been found in the US [1, 3, 17, 20, 33], and the preferred techniques have also been found to vary internationally as well [8].

The distribution of the operative techniques, as depicted in Table 2, differs from the recently reported practices in the state of New York, where posterior or even circumferential fusion surgery was done for 5.4% of patients diagnosed with degenerative disc and undergoing cervical fusion surgery [26]. The choices of approach and technique must take into consideration many anatomical and patient-related factors, such as the disc height, the extent of the degenerative fusion, the alignment of the vertebrae, the direction of the compression, the number of affected levels and possible instability as well as the age and the comorbidities of the patient [34, 35]. The evidence comparing the different surgical approaches and techniques is weak and conflicting, very likely reflecting the variability of the clinical scenarios [4–6, 9–13, 16, 28, 30]. The lack of robust evidence on the best technique for many degenerative problems allows for differences in the treatment practices. For degenerative cervical myelopathy, anterior surgery is more common in Europe in comparison with Latin America or North America [8]. Posterior decompressions have internationally moved from laminectomies to either laminoplasty or PDF because of the risk of post-laminectomy kyphosis [21, 25]; in Finland, the incidence of PDF for DCSD has remained moderate. Based on the NOMESCO codes, we were not able to differentiate between laminectomy and laminoplasty. However, the latter is by tradition seldom used for SCS on adult patients in Finland. The cost and the complication rate of PDF are higher than the anterior approaches [27], which is likely to influence the operative choices. Further, the evidence to support the standard use of fusion in conjunction with posterior decompression is not very strong [2, 21, 25]. The mean age of the patients operated in Finland did not differ from the US [19, 23], but there may still be differences in the prevalence or the extent of degeneration [8], which could affect the operative techniques used [20, 32]. The way in which reoperations and complications such as kyphosis, instability or inadequate decompression are handled in Finland may also play a role in the choice of the operative techniques. The patients with healthcare-related injuries are compensated jointly through the Finnish Patient Insurance Centre, and the compensation is aligned with the compensation received for similar injuries acquired in an accident. This, in conjunction with a fixed salary rather than a salary based on the number of operations a surgeon performs, may lessen the incentive to perform instrumentation or extensive surgery. The financial interests of surgeons have been offered as one potential explanation for the increasing operation rates and fusion rates in the United States [3, 23]. There may also be differences in the relief of the symptoms, complications or reoperation rates, which were not analysed here.

### Strengths and limitations of the study

Every patient who was operated on and fulfilled the inclusion and the grouping criteria was included in the study from all the hospitals in Finland. The exclusion of the reoperations was reliable due to the use of the PICs. The adjustment for age and sex was reliable as the public records are precise and include every inhabitant. The selection bias inherent in retrospective studies was probably low. No posterior fusions were recorded in the disc protrusion group, and only PDFs were recorded in the AAS group, which corroborates the quality of the data.

We defined the diagnostic and the procedure groups by using the ICD-10 and the NOMESCO coding systems. The NOMESCO coding does not fully comply with the developing operative techniques, and the codes traditionally used vary between hospitals. The operative code for foraminotomy was also used for ACDF in Helsinki between 1999 and 2006, which influences the operative incidences of ACDF and decompression procedures prior to 2007. Accordingly, 688 patients with diagnostic codes classified as spinal canal stenosis had the operative code for foraminotomy (ABC30) and were classified as spinal canal stenosis operations. In the other four hospitals, the fusion codes (NAG40/41 or ABC21) were used for ACDF during the entire period studied (personal communication). To investigate the effect of the coding practice change, we analysed separately the proportional use of the different techniques in each of the university hospitals (Fig. 5). There may also be other discrepancies in the codes used that may influence the results and certainly affect the analysis of the use of the specific operation techniques, as evidenced by the differences in the distribution of the techniques among the hospitals (Supplementary Table S2). We feel such discrepancy is the product of differences in coding practices rather than actual operative techniques.

Due to the imprecision of the NOMESCO coding, it is not possible to differentiate between the anterior and the posterior decompressions with certainty, but for the fusions, the codes are reliable. For decompression procedures, a differentiation is partially made for open, microsurgical and endoscopic procedures on the discs and the neural foramina. For fusions, anterior and posterior operations can be identified, but discerning the number of levels fused or the inclusion of the occiput in the fusion is not possible. The registered information on the use of plating in conjunction with anterior decompression is also unreliable due to the variance in the use of the NOMESCO codes. Therefore, precise information on the use of plates also cannot be gleaned from the data.

Many of the other weaknesses in our study are typical for analyses utilising administrative databases. Most importantly, significant clinical information, such as the number of affected vertebral levels, the distribution and the extent of the degenerative changes, the alignment of the vertebrae or the clinical symptoms (i.e. radicular/myelopathic/axial neck pain) cannot be determined from the administrative data. These are factors that strongly affect the choice of the operative approach and technique [34, 35].

### Generalisability

We have included all the primary operations performed in Finland over the study period, with a presumably low selection bias. These results represent trends that are independent of the changes in the population age or sex distribution as well as the surgeon income or the insurance coverage. The operative technique is decided by the surgeon and the patient without the influence of the payer. Therefore, the trends portray the perceived best practice in every hospital.

### Conclusions

Between 1999 and 2015, the operative techniques used for DCSD changed from prevalently decompressive to include fusion in 75% to over 90% of the operations, depending on the treating hospital. ACDF became the most commonly applied technique for all the degenerative diagnoses except AAS and in all but the oldest age group. The incidence of PDF declined due to a decline in surgery for rAAS. The use of PDF for DCSD also increased in Finland, but the incidence is only approximately 10% of the reported PDF incidences in the US, most likely due to differences in operation indications as well as financial influences. The change in the operative techniques utilised in Finland was in all probability based on clinical experience and perhaps the emergence of industrially manufactured cages, as the few randomised controlled trials have not shown clinically relevant differences in the outcomes between the techniques. While there may indeed be no differences in the outcomes between the techniques, this is more likely due to the heterogeneity of the degenerative changes and the clinical presentation. The techniques, without solid scientific support, will continue to evolve towards more invasive and expensive procedures unless randomised controlled trials are conducted separately analysing the defined diagnostic groups.

## Electronic supplementary material

ESM 1 (PDF 212 kb)

## Data Availability

The data can be attained from the National Institute for Health and Welfare via the corresponding author upon justifiable request.

## **Abbreviations**

ACDF: anterior cervical decompression and fusion
AAS: atlanto-axial subluxation
DCSD: degenerative cervical spine disease
FHDR: Finnish Hospital Discharge Register
FS: foraminal stenosis
ICD: International Classification of Diseases
NOMESCO: Nordic Medico-Statistical Committee Classification of Surgical Procedures
PDF: posterior decompression and fusion
PERFECT: Performance, Effectiveness and Cost of Treatment Episodes
PIC: personal identity code
RA: rheumatoid arthritis
rAAS: rheumatoid atlanto-axial subluxation
SCS: spinal canal stenosis
SII: social insurance institution
TDR: total disc replacement
US: United States
WHO: World Health Organization
