# Supplementary material for: Surgical techniques for degenerative cervical spine in Finland from 1999 to 2015
Source: Acta Neurochir (Wien). 2019 Aug 10;161(10):2161–73. doi: 10.1007/s00701-019-04026-9 (PMC6739280; doi:10.1007/s00701-019-04026-9)
Supplement: Supplementary file 1 — (PDF 212 kb) [file 701_2019_4026_MOESM1_ESM.pdf]

Supplementary material to "Surgical techniques for degenerative cervical spine in Finland from 1999 to 2015".

Supplementary Table S1. Adjusted annual incidence of posterior decompression and fusion operations in each diagnostic group (operations / 100 000 people age 18 or older).

Year	Disc protrusion	Foraminal stenosis	Spinal canal stenosis	Degenerative atlanto-axial subluxation	Degenerative indications combined
**1999**	0	0	0	0.1	0.2
**2000**	0	0.1	0	0.1	0.2
**2001**	0	0.1	0.1	0.1	0.2
**2002**	0	0	0.1	0.1	0.2
**2003**	0	0.1	0.1	0.1	0.3
**2004**	0	0	0.3	0.1	0.4
**2005**	0	0	0.2	0	0.3
**2006**	0	0.1	0.2	0	0.3
**2007**	0	0.1	0.4	0	0.5
**2008**	0	0.1	0.2	0	0.3
**2009**	0	0.2	0.3	0	0.4
**2010**	0	0.3	0.3	0	0.6
**2011**	0	0.1	0.5	0	0.6
**2012**	0	0.1	0.5	0	0.6
**2013**	0	0.3	0.3	0	0.6
**2014**	0	0.2	0.5	0.1	0.7
**2015**	0	0.2	0.4	0	0.7
**Overall**	0	0.1	0.3	0	0.4

Supplementary Table S2. The distribution of operative techniques according to the NOMESCO[a] operative codes in each university hospital.

Operative techniques		The distribution of the operations in each hospital, % (N)					
		Helsinki	Kuopio	Oulu	Tampere	Turku	Overall
**Anterior decompression**	**ABC01 ABC10 ABC20**	1.5 (95)	1.0 (50)	3.4 (73)	2.4 (112)	7.3 (132)	2.3 (461)
**Foraminotomy***	**ABC30**	30.0 (1876)	3.2 (158)	17.4 (347)	10.8 (498)	6.8 (122)	15.2 (3001)
**Unspecified decompression**	**ABC99**	5.9 (370)	0.9 (46)	0.7 (15)	2.2 (102)	1.1 (20)	2.8 (553)
**ACDF[b] without plating**	**NAG40 ABC21**	44.2 (2764)	82.1 (4041)	61.6 (1303)	60.0 (2772)	8.3 (150)	56.0 (11030)
**ACDF[b] with plating***	**NAG41**	3.4 (215)	3.7 (180)	4.2 (88)	12.8 (590)	52.1 (937)	10.2 (2010)
**Disc prosthesis**	**NAB92**	0.4 (22)	0.0 (1)	0.9 (19)	0.1 (3)	0.7 (12)	0.3 (57)
**Corpectomy**	**NAG72**	0	0	0.1 (2)	0.0 (1)	0.1 (1)	0.0 (4)
**Laminectomy / laminoplasty**	**ABC50 ABC60**	12.3 (771)	6.2 (306)	9.5 (202)	8.5 (391)	17.4 (313)	10.1 (1984)
**Posterior decompression and fusion**	**NAG42**	2.1 (134)	2.8 (138)	3.2 (67)	3.3 (151)	6.2 (111)	3.1 (601)
**Total**		6247	4920	2116	4620	1798	19701
* The foraminotomy code (ABC30) was used for ACDF as well as foraminotomy in Helsinki until 2005-2006; the ACDF code NAG41 was used in Turku for most ACDF operations, with or without plating							

[a]NOMESCO Nordic Medico-Statistical Committee Classification of Surgical Procedures, [b]ACDF Anterior cervical decompression and fusion

Supplementary Table S3. The distribution of the operative techniques within the diagnosis groups (%, N).

	Anterior decompression	Foraminotomy*	Unspecified decompression	ACDF[b] without plating	ACDF[b] with plating*	Disc prosthesis	Corpectomy	Laminectomy / laminoplasty	PDF[c]
**Disc protrusion**	6.7 (460)	14.9 (1029)	0	59.7 (4134)	16.5 (1141)	0.8 (57)	0	1.5 (103)	0
**Foraminal stenosis**	0	19.0 (1306)	0.6 (40)	72.8 (5001)	5.6 (383)	0	0	0.8 (56)	1.3 (88)
**Spinal canal stenosis**	0	11.9 (666)	9.2 (513)	34.0 (1895)	8.7 (486)	0	0.1 (4)	13.8 (773)	3.4 (192)
**Rheumatoid AAS[a]**	0	0	0	0	0	0	0	0	100.0 (30)
**Degenerative AAS[a]**	0	0	0	0	0	0	0	0	100.0 (291)
* The foraminotomy code (ABC30) was used for ACDF as well as foraminotomy in Helsinki until 2005-2006; the ACDF code NAG41 was used in Turku for most ACDF operations, with or without plating									

[a]AAS Atlanto-axial subluxation, [b]ACDF Anterior Cervical Decompression and Fusion, [c]PDF posterior decompression and fusion

Supplementary Figure S4. The proportional use of anterior (a) and posterior (b) techniques in the treatment of degenerative cervical spine disease between 1999 and 2015.

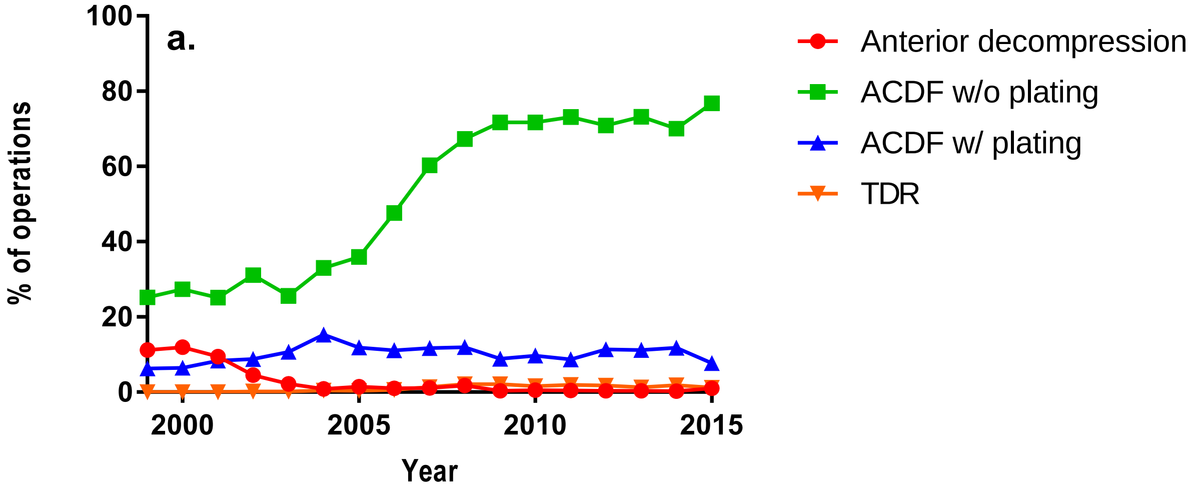

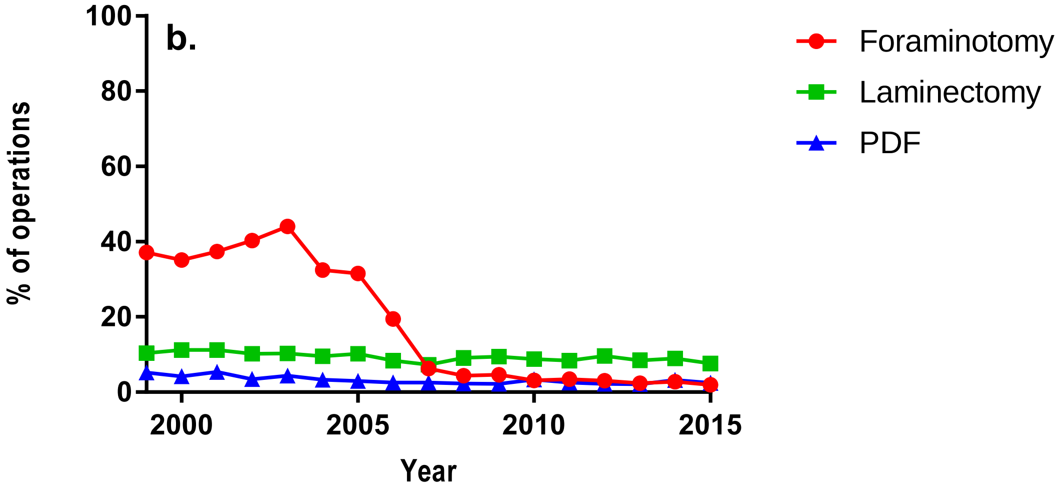

ACDF Anterior cervical decompression and fusion, w/ with, w/o without, TDR total disc replacement, PDF posterior decompression and fusion.
